# Technique and results after immediate orthotopic replantation of extracorporeally irradiated tumor bone autografts with and without fibular augmentation in extremity tumors

**DOI:** 10.1186/s12891-021-04629-3

**Published:** 2021-08-31

**Authors:** Alexander Klein, Yasmin Bakhshai, Falk Roeder, Christof Birkenmaier, Andrea Baur-Melnyk, Hans Roland Dürr

**Affiliations:** 1 Musculoskeletal Oncology, Department of Orthopaedics, Physical Medicine and Rehabilitation, University Hospital, Marchioninistr. 15, 81377 Munich, Germany; 2 Department of Orthopaedics, Physical Medicine and Rehabilitation, University Hospital, LMU, Munich, Germany; 3 Department of Radiotherapy and Radiation Oncology, Landeskrankenhaus, Paracelsus Medical University, Salzburg, Austria; 4 Department of Radiology, University Hospital, LMU, Munich, Germany

**Keywords:** Irradiated bone, Bone reconstruction, Bone tumor, Fibular graft, Pseudarthrosis, Autograft

## Abstract

**Background:**

Reconstruction of the skeletal defects resulting from the resection of bone tumors remains a considerable challenge and one of the possibilities is the orthotopic replantation of the irradiated bone autograft. One technical option with this technique is the addition of a vital autologous fibular graft, with or without microvascular anastomosis. The aim of our study was to evaluate the clinical results of the treatment of our patient cohort with a specific view to the role of fibular augmentation.

**Methods:**

Twenty-one patients with 22 reconstructions were included. In all cases, the bone tumor was resected with wide margins and in 21 of them irradiated with 300 Gy. In the first case, thermal sterilization in an autoclave was used. The autograft was orthotopically replanted and stabilized with plates and screws. Fifteen patients underwent an additional fibular augmentation, 8 of which received microvascular anastomoses or, alternatively, a locally pedicled fibular interposition.

**Results:**

the most common diagnosis was a Ewing sarcoma (8 cases) and the most common location was the femur (12 cases). The mean follow-up time was 70 months (16–154 months). For our statistical analysis, the one case with autoclave sterilization and 3 patients with tumors in small bones were excluded. During follow-up of 18 cases, 55.6% of patients underwent an average of 1.56 revision surgeries. Complete bony integration of the irradiated autografts was achieved in 88.9% of cases after 13.6 months on average. In those cases with successful reintegration, the autograft was shorter (n.s.). Microvascular anastomosis in vascularized fibular strut grafts did not significantly influence the rate of pseudarthrosis.

**Conclusions:**

the replantation of extracorporeally irradiated bone autografts is an established method for the reconstruction of bone defects after tumor resection. Our rate of complications is comparable to those of other studies and with other methods of bone reconstruction (e.g. prosthesis). In our opinion, this method is especially well suited for younger patients with extraarticular bone tumors that allow for joint preservation. However, these patients should be ready to accept longer treatment periods.

## Background

Most primary malignant bone tumors are localized in the long bones with the majority occurring in the meta−/epiphyses whereas about 10% affect the diaphyseal part of the long bones [1]. The resection of these tumors causes large defects in these load-bearing bones and their reconstruction represents a challenge. Biological as well as endoprosthetic methods for the reconstruction of long bone continuity have been developed. Biological reconstruction methods include the use of allografts, segmental transport or the reimplantation of sterilized resected bone segments [2] with each of these methods entailing specific advantages and disadvantages. The long-term problems are infection, graft necrosis, implant loosening or pseudarthrosis. The reimplantation of a sterilized autograft offers the advantages of a perfect anatomical match and immediate availability. The available methods for the devitalization of the bone autograft are sterilization by irradiation, deep-freezing using fluid nitrogen and pasteurization [2]. Our center has a longstanding experience with the sterilization of tumor-bearing bone autografts by irradiation. This technique was first described by Spira et al. in 1968 [3] and has since become established in the treatment of malignant bone tumors. It allows for an anatomic reconstruction and later bone remodeling especially in young patients [4]. However, there are only few reports with series including more than 15 patients.

There are two major problems with this strategy: One is the creeping resorption of the autograft, the other is the reported high rate of pseudarthroses. In order to combat these issues, the augmentation of the irradiated autograft with a vascularized or a non-vascularized fibula segment is an option. Until now, the role of fibular augmentation of an irradiated autograft has not been fully established.

## Methods

The aim of our study was to evaluate the outcomes of the treatment of malignant bone tumors by replantation of the extracorporeally irradiated bone segments, to establish the rate of successful bone healing and to evaluate the role of fibular augmentation (vascularized or non-) of the reconstruction. Additional factors, which might influence the result, were also analyzed.

This retrospective analysis was performed based on our tumor database and current follow-up data. We identified 21 consecutive patients with 22 resections, irradiation and replantation of the tumor bone segment, operated between 1999 and 2015. The same surgeon treated all patients. The indications for this procedure were the following:

- Bone defect after wide resection of a tumor
- Localization of the tumor in the long bone
- Sufficient structural stability of the segment to be resected and replanted

The diagnosis of the tumor was ascertained by an incisional or a core-needle biopsy based on radiological imaging (magnetic resonance imaging (MRI), computed tomography (CT)- and/or positron emission tomography-computed tomography (PET-CT) scan). Primary bone tumors as well as metastases of other tumor entities were included. Systemic therapy was applied in some patients depending on the requirements of the underlying tumor condition. The indication for wide resection was either a primary bone tumor or metastatic disease in selected cases of cancers less sensitive to radiation (e.g. renal cell carcinoma) as a curative approach.

There were 9 female and 12 male patients. The range of the age at the time of operative treatment was between 10 and 83 years (median age 36.4 years), 3 of them younger than 18 years. These 21 patients underwent 22 primary operations. One patient had a simultaneous partial femur and tibia resection. No patient was lost to follow-up.

Systemic therapy was indicated in respect to the entity of the tumor. The patient with chondrosarcoma did not receive any systemic therapy. Patients with Ewing and osteosarcomas underwent standardized chemotherapy in neoadjuvant and adjuvant settings: Euro-EWING regime for Ewing sarcomas [5]; EURO-B.O.S.S [6]. or EURAMOS [7] regime for osteosarcomas. The indication for resections of metastatic disease was a resectable lesion of the bone in selected entities as renal cell or hepatocellular carcinoma known to be less sensitive to radiotherapy. In general, patients were free of tumor after resection of the primary tumor and metastatic disease; there was no indication for systemic treatment. After wide resections of the tumor with clear margins, no indication for radiotherapy was seen. The only exception had been patients with Ewing sarcoma. In those cases, an adjuvant radiotherapy was discussed individually.

For sterilization we used high-dose irradiation in all but the first patient, where thermal sterilization in an autoclave was used.

In the case of fibular augmentation, harvesting the graft was the first step of surgery. The length of the fibular graft was calculated to cover both osteotomy sites. A wide resection of the tumor was subsequently performed. The explanted tumor-bearing bone segment was packed into a double sterile bag and transported to the radiation oncology department. To minimize any build-up effect and to keep radiation time short, the bag was wrapped with flab material and placed beneath the linear accelerator with the lowest possible distance to the accelerator head, usually on a tray in the accessory slot. A dose of 300 Gy (Gy) using an opposing field technique was applied in a single fraction. After radiation, the bone (fragment) was immediately returned to the operation room. During sterilization, we obtained biopsies of the bone marrow tissue from the ends of the remaining bone and from surrounding soft tissue for the evaluation of the resection margins.

**Fig. 1:**
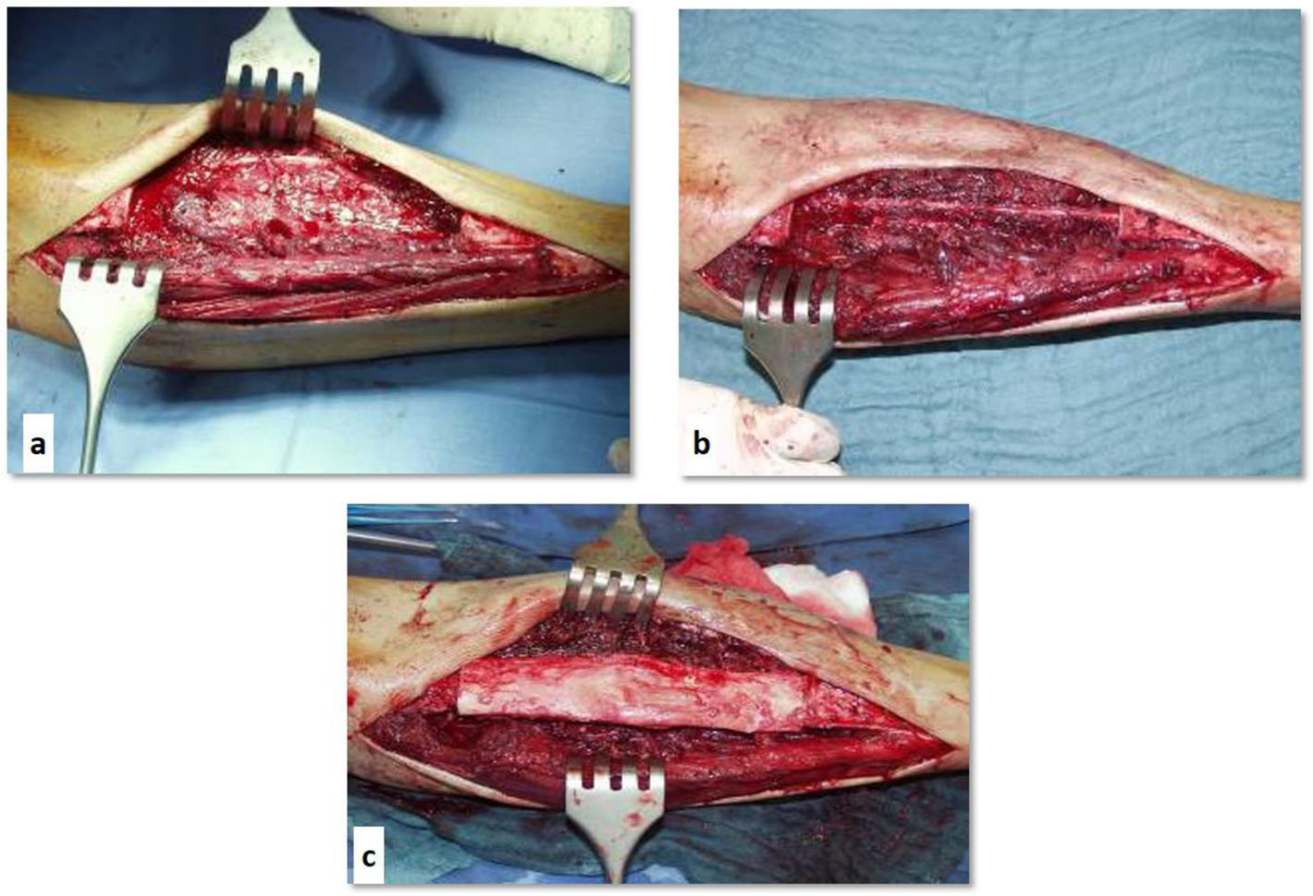
Technique of tumor resection and biological reconstruction with irradiated diaphyseal tibia autograft. **a**: Resection of the tumor in the tibial diaphysis; **b**: Transposition of the ipsilateral fibula into the tibial defect; **c**: Interposition of the irradiated segment covering the fibular transplant prior to osteosynthesis. The plate osteosynthesis (Limited Contact Dynamic Compression Plate) was performed as next step

**Fig. 2:**
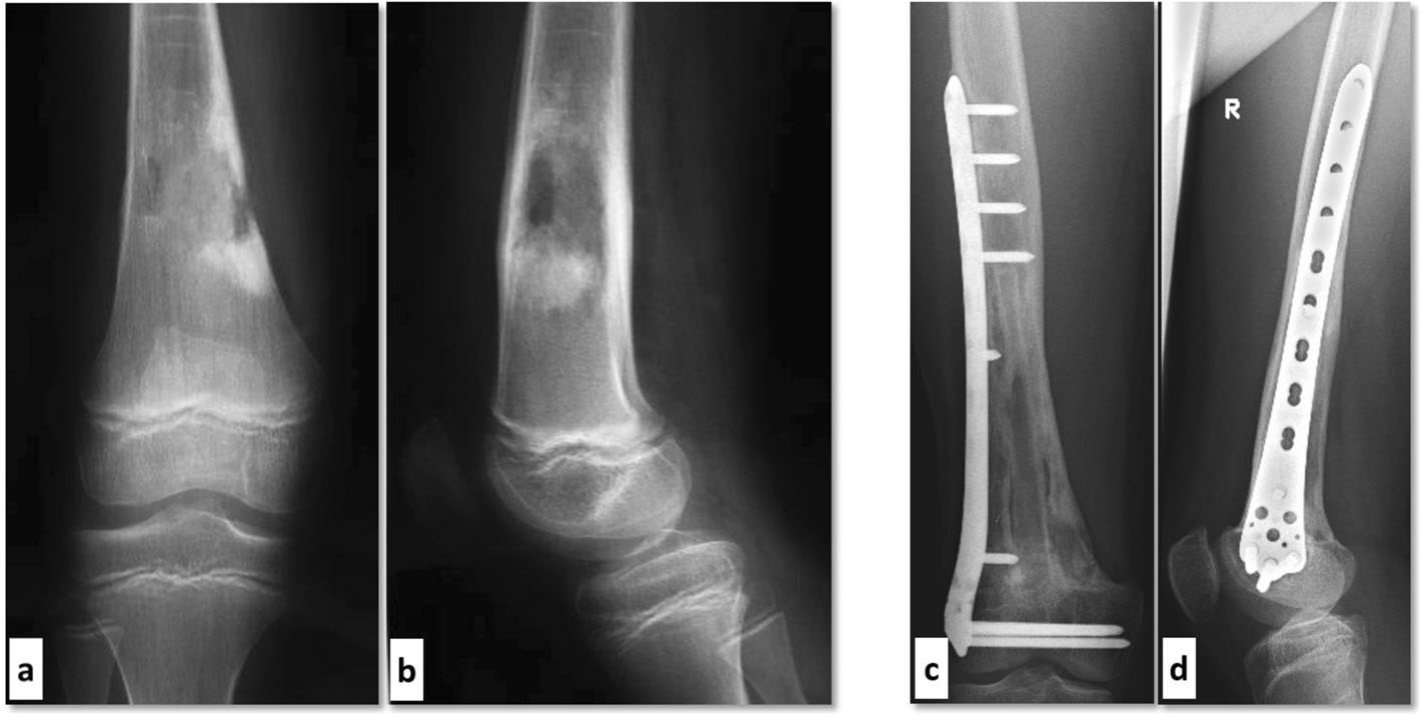
Osteosarcoma of the distal femur in an 11-year-old boy; **a** + **b**: Preoperative a.p. and lateral view; **c** + **d**: Bony integration using a non-vascularized fibular augmentation 4 years after surgery

In the next step, the irradiated bone was prepared for replantation, the soft tissue parts were resected as necessary (Fig. 1), followed by replantation and osteosynthesis. The fibular graft was intramedullary positioned inside the sterilized autograft (inlay technique) covering both osteotomies. In 15 patients, an augmentation with a fibular graft was used. In 8 of these 15 cases, we used a vascularized fibula or performed a pedicled fibular interposition. The decision, which method (vascularized/non-vascularized) fibula was used, depended primarily on the location. If a fibular transposition into a tibial defect was possible, we used a pedicled vascularized graft. In the first cases of femoral defects, we used free vascularized fibula grafts. Later, due to good reported experiences with non-vascularized fibula graft in literature [8], we changed our strategy and used non-vascularized grafts. The reconstruction was stabilized in long bones with one long plate osteosynthesis with anchoring proximal and distal of the osteotomy (regularly with locking screws). In most cases, the replanted graft was fixed in the plate with one monocortical screw (Fig. 2). The rehabilitation included complete non-weight bearing for 6 weeks postoperatively and gradual increase of the load of the affected extremity in dependence of the radiographic follow-up. The conventional radiographic controls were performed 6 weeks, 3, 6, 9 and 12 months after the surgery and then as required. Full weight-bearing could be achieved in general after 10–12 weeks. The local tumor follow-up was done by means of MRI. The presence or absence of bony union was assessed by an experienced musculoskeletal radiologist based on the conventional radiographs obtained at follow-up (Fig. 2).

Significance analysis was performed using the Log-Rank test or the Chi-Square test, defining a 95% confidence interval. The univariate analysis (Cox proportional-hazards regression) was used for the evaluation of the influence of the distance of bone resection and of the reconnection of fibular vessels on the pseudarthrosis rate. Because of the small cohort size, we re-evaluated the significance with the Fisher exact test. The level of significance was set at less than 0.05. The data analysis software used was IBM® SPSS® Statistics 25. The institutional ethics committee approved this study.

## Results

All patients with replantation of irradiated autografts could be included. One patient underwent synchronous dual tumor resection und replantation of irradiated bone in the distal femur and in the proximal tibia for bone metastases of a renal cell carcinoma.

The diagnoses were Ewing sarcoma in 8 cases, metastatic disease in 6 cases (5 patients with renal cell carcinoma, one patient with hepatocellular carcinoma), osteosarcoma in 5 and one case each with high-grade chondrosarcoma, leiomyosarcoma and myxofibrosarcoma with bone involvement (Table 1). The most common site of the tumors was the femur in 12 cases, the tibia in 7 and the calcaneus in 2 cases. One patient had the tumor located in the scapula. The mean follow-up was 70 months (range between 16 and 154 months, median 58 months). In only 3 patients the follow-up was less than 2 years. In total, five patients deceased due to progressive disease during the observation period. 2.54 revisions per case were necessary in the whole cohort (range between 0 and 8).We divided the cohort in two groups of patients: 18 cases with tumor location in the long bones and 3 patients with involvement of small/flat bones (2 cases with calcaneus and 1 case with scapula tumor). Also the result of one case with an autoclaved autograft is described independently.

**Table 1:** Data of patient cohort and details of surgery (sex: m = male, f = female; HCC: hepatocellular carcinoma; RCC: renal cell carcinoma)

Pat ID	Age	Tumor Entity	Location	Site of proximal osteotomy	Site of distal osteotomy	Fibula-augmentation	Type of complication	Number of revisions	Bony union achieved
Location in long bones									
1	10	Ewing Sarcoma	tibia	metaphysis	diaphysis	yes/vascularized	infection	5	yes
2	11	Osteosarcoma	femur	diaphysis	metaphysis	yes/vascularized		0	yes
3	66	Osteosarcoma	femur	diaphysis	metaphysis	no	pseudarthrosis	2	yes
4	57	Leiomyosar-coma	tibia	metaphysis	diaphysis	yes/vascularized		0	yes
5	26	Ewing Sarcoma	femur	diaphysis	diaphysis	yes/non-vascularized		0	yes
6	13	Osteosarcoma	femur	metaphysis	metaphysis	yes/non-vascularized	pseudarthrosis	4	prosthesis
7	58	HCC-Metastasis	femur	metaphysis	epiphysis including cartilage	no	infection	2	yes
8	27	Ewing Sarcoma	femur	metaphysis	diaphysis	yes/non-vascularized		0	yes
9	37	Osteosarcoma	femur	diaphysis	diaphysis	yes/vascularized	pseudarthrosis	8	yes
10	79	RCC-Metastasis	tibia	diaphysis	epiphysis including cartilage	no	hematoma	1	yes
11	83	RCC-Metastasis	femur	diaphysis	metaphysis	no	pseudarthrosis	1	yes
12	62	Osteosarcoma	tibia	diaphysis	diaphysis	yes/non-vascularized		0	yes
13	12	Ewing Sarcoma	femur	metaphysis	diaphysis	yes/vascularized	pseudarthrosis	1	pseudarthrosis
14	73	RCC-Metastasis	femur	diaphysis	metaphysis	yes/non-vascularized		0	yes
		RCC-Metastasis	tibia	metaphysis	diaphysis	yes/vascularized	infection	3	yes
15	31	Chondrosar-coma	tibia	epiphysis including cartilage	metaphysis	no		0	yes
16	68	Myxofibrosar-coma	femur	diaphysis	metaphysis	yes/non-vascularized	pseudarthrosis	1	yes
17	49	RCC-Metastasis	femur	diaphysis	metaphysis	yes/non-vascularized		0	yes
Patient with autoclaved autograft									
18	20	Ewing Sarcoma	tibia	metaphysis	diaphysis	yes/vascularized	wound healing	2	yes
Location in short/flat bones									
19	36	Ewing Sarcoma	calcaneus	metaphysis		no	wound healing	2	yes
20	32	Ewing Sarcoma	scapula		metaphysis	no	hematoma	1	pseudarthrosis
21	29	Ewing Sarcoma	calcaneus			no		0	not possible (total bone)

### Tumor control

We observed only one locoregional recurrence in 21 patients, resulting in a local control rate of 95.5%. This patient, a 13-year old boy, suffered from an osteosarcoma of the femoral diaphysis. He had been diagnosed with a pathologic fracture surrounded by a large hematoma and had initially been treated by elastic intramedullary nailing at another institution. The hip and the knee joint were not contaminated. Two surgical options after neoadjuvant chemotherapy were discussed with the patient and his parents: total femur resection and endoprosthetic reconstruction or resection of the contaminated part of femur and biological reconstruction. We subsequently performed a resection with microscopically clear margins and replantation as described in the methods section. He developed a symptomatic pseudarthrosis between the graft and the femur, which was salvaged by endoprosthetic total femur reconstruction. Nine months after endoprosthetic reconstruction, locoregional recurrence occurred (outside the initial graft area but inside the initial hematoma area), which was successfully salvaged by wide resection. We therefore attribute the locoregional recurrence to the initial fracture hematoma, which most probably had been contaminated with tumor cells. The patient is free of tumor for now 14 years.

#### Patients with long bone tumors

There were 11 tumors in the diaphyseal segment of the bone. Seven lesions were located in the epi−/ metaphyseal part of the bone. Out of 18 cases of replantations in long bones, 15 osteotomies were located in the metaphyseal zone, 18 in the diaphyseal area. Three patients had a replantation of a meta-epiphyseal graft including the articular cartilage (Table 1). When comparing the time required the osteotomy to heal between diaphyseal and meta-epiphyseal sector, no significant difference was observed (*p* = 0.662; 10.8 vs 11.4 months). Comparing the proximal and distal osteotomies, there was no significant difference in rate of pseudarthrosis (*p* = 0.336).

The length of the resected intercalary segment was not significantly correlated with the occurrence of pseudarthrosis (*p* = 0.229), but the cases with a pseudarthrosis had longer resections lengths on average (16.9 cm vs 12.7 cm).

In 13 cases, we performed a fibular augmentation Two pseudarthroses were observed after reconstruction without and 4 after reconstruction with fibular augmentation. The use of fibula strut grafts for augmentation of the autograft did not improve the pseudarthrosis rate, compared to non-augmented reconstructions (*p* = 0.561). 7 of 13 fibular autografts were non-vascularized. There was one case of pseudarthrosis in the group of vascularized and three in the group of non-vascularized augmentations. In our statistical analysis, however, there was no significant difference between these groups (*p* = 0.343). With a microvascular anastomosis, the time to bony integration was 11.7 months on average (median 10.5; range 9–16 months) and 13 months in the non-vascularized group (median 10; range 8–27 months; *p* = 0.712, Fisher exact test *p* = 0.396). The results of univariate analysis are shown in the Table 2.

**Table 2:** Results of univariate analysis regarding the risk of pseudarthrosis in cases involving the long bones

	*p*-value
Location in bone (diaphyseal vs. meta−/epiphyseal)	0.662
Location in bone (proximal vs. distal osteotomy)	0.336
Length of the resection	0.229
Use of fibular graft	0.561
Vascularization of fibula graft	0.343

### Graft integration

The results from 18 cases with such reconstructions were evaluated. (Table 1). Complete bony integration of the irradiated autograft was ultimately achieved in 16 of 18 lesions (88.9%) after a mean time of 13.6 months (range 4–35, median 10 months) and – in several instances – revisions for initial non-union (see below). In total, 6 patients with long bone reconstruction developed pseudarthrosis, 4 united, one necessitated an endoprosthesis (because of local recurrence) and one remained asymptomatic and did not require any further surgical treatment. 4 of 6 pseudarthrotic osteotomies were located in metaphyseal and also four of 6 in the proximal part of the bone. From all symptomatic pseudarthroses the failure of osteosyntheses did cause the pain. We used for revision autologous spongiosa from the iliac crest and re-osteosynthesis with a plate. Between 1 and 8 revisions (on average 3 per case) were necessary in cases of pseudarthroses. Limb preservation was achieved in all patients.

### Complications

We observed complications that required surgical revision in 10 out of 18 cases (55.6%). 6 of those were caused by symptomatic pseudarthrosis with failure of the reconstruction and led to at least one surgical revision per patient. Three Patients needed an additional operation because of a surgical site infection and 1 because of wound hematoma 3. In total, 28 revisions were necessary (average 1.56 revisions per case). There were no cases of autograft fracture (Table 1).

#### Patients with reconstructions at the small/flat bones or after autoclaving

Patient 1.

20-year-old patient with recurring Ewing sarcoma of the proximal tibia. She was the first patient in our cohort. In this case only we used thermal sterilization in an autoclave. We used the ipsilateral fibula transposition for the augmentation of the reconstruction. Two surgical revisions 28 days after the first surgery were caused by wound necrosis. We achieved complete wound healing by meshed skin transplantation. The bone osteotomy healing was successful 11 months postoperatively.

### Patient 2

32-year-old patient with Ewing sarcoma of the scapula. After subtotal resection leaving a small part with the glenoid fossa, the autograft was replanted and fixed by plate osteosynthesis. Revision on the fifth postoperative day was necessary because of hematoma. The osteotomy developed a stable pseudarthrosis, the function of the shoulder is satisfactory after 11 years of follow-up.

### Patient 3

29-year-old patient with Ewing sarcoma of calcaneus. After total resection and irradiation of the bone, augmentation with bone cement and replantation was performed. Full weight bearing and free walking was achieved 15 months after surgery. This patient died 2.5 years after surgery due to pulmonary metastases.

### Patient 4

36-year-old patient with Ewing sarcoma of calcaneus. After partial resection of the dorsal part of calcaneus and irradiation, the graft was augmented with bone cement and osteosynthesis was performed (screws). Two revisions were necessary because of wound healing impairments within the first 4 weeks. After wound healing, the patient was able to walk free without local pain and is alive 6.5 years after surgery.

## Discussion

The reconstruction of bone defects is not a trivial task and several strategies are available: endoprostheses, allografts, autologous fibula grafts, allogeneic bone grafts, bone segment transfer and sterilized autologous grafts.

The option of reconstruction by means of megaprostheses allows for the rapid stabilization of the affected bone or joint. Full weight bearing and predictable good function of the extremity can be achieved rapidly after bony integration of the stems within 4–8 weeks. However, there are only few reports on long-term results with these megaprostheses, but for intercalary reconstruction, they have a loosening rate of 25% within a rather short follow-up time of 14 months. The most critical location appears to be the femur [9]. In endoprosthetic joint replacements, Grimer et al. presented their results with a mean follow-up of 29 years. Every patient required on average 2.7 further operations during the follow-up period with the risk of infection being 1% per year of life and every further operation increasing the infection risk by 2.7%. The risk of secondary amputation was 16% [10]. Our revision rate (2.54 per case) is comparable with that reported for endoprosthetic reconstruction. Because of the long-term results observed by Grimer et al. and an increasing risk of complications over time, the option of biological reconstruction becomes more important especially for young patients. The long-term observation of patients after biological reconstruction by irradiated autografts confirms this thesis [11].

One of the latest published studies presents the results of 64 patients with allograft reconstruction of a resection defect in the lower extremity. The overall survival of the reconstructions was 90% after 15 years. 6% of patients underwent secondary amputation of the limb. At least one surgical revision was needed in 40.6% of patients [12]. Especially the diaphyseal reconstructions seemed to be difficult: 70% of these patients needed an operative revision and in 40% of them, non-union was the reason for the reoperation [13].

The rate of revision surgeries in our series was 59%. Pseudarthrosis was the primary reason for revision in 27% of patients (46% of all reoperations). Despite this, the osteotomy healing time was comparable between diaphyseal and other locations. The diaphyseal location appears to have a higher failure rate of the mechanical reconstruction in the early phase of healing because of the high mechanical load on a small bone diameter. One possible explanation is the differences in bone biology and in mechanical behavior between these bone parts [14].

Because of the most common complication being pseudarthrosis, we analyzed the factors that could have influenced the outcome. In our patient cohort, we were unable to identify any predictive factors for a successful osteointegration. The length of the reimplanted bone segment within the group with failed reconstructions was longer, but at the same time, this is a factor, which is determined by the tumor extent. The literature contains more work on the role of fibular autograft interposition with or without vascular supply. The role of vascular supply is not yet finally clarified. Manfrini at al could not show a positive influence of pedicled fibula autograft on the surgical revision rate (incl. pseudarthrosis) [15]. Other publications showed an advantage of the vascularized fibula in the case of an irradiated tumor bed or of perioperative chemotherapy [16]. The extensive review by Allsopp et al. in 2016 found no advantages with vascular reconnection of fibular grafts. On the other hand, their work provides evidence of an even higher complication rate with vascularized autografts [17].

The type of sterilization of the bone graft also appears to have an influence on the healing capacity of the so treated bone. One experimental series demonstrated the lowest rate of pseudarthrosis after the replantation of irradiated bone in comparison to pasteurized or autoclaved bone [18]. We used the technique of autograft sterilization by irradiation. The radiation dose necessary to guarantee necrosis of tumor tissue with extracorporal irradiation was calculated to be 250 Gy [19]. In the published literature, an application of radiation dosages between 50 to 300 Gy are described. We used 300 Gy to be on the safe side with respect to tumor necrosis. The negative effects of irradiation on bone biology are well established [20–22]. We did not observe any cases of autograft fracture in our cohort. For irradiated grafts and using a low dose of irradiation, the cadaver study by Hernandez et al. did not show any influence on the biomechanical properties of the irradiated bone [23]. These findings might explain to a large extent the reconstruction failures and minimally longer time of osteotomy healing in our cohort, compared to other studies [24, 25]. Further studies would be required to further evaluate the influence of the irradiation dose on the clinical result after the bone reconstruction by irradiated autograft.

## Conclusion

One established method to reconstruct bone defects after tumor resection is the replantation of the irradiated autograft. The rate of complications with this method in our hands is comparable to previously published studies and to alternative methods of bone reconstruction (e.g. prostheses). In our opinion, this method is best suited for young patients with extraarticular bone tumors. However, these patients should be ready to accept longer treatment periods.

## Data Availability

The datasets used and/or analysed during the current study are available from the corresponding author on reasonable request.

## Abbreviations

MRI: magnetic resonance imaging
CT: computed tomography
PET-CT: positron emission tomography-computed tomography
Gy: gray
cm: centimeter
